# SOCIAL ENGAGEMENT AMONG OLDER PEOPLE IN ENGLAND SINCE THE COVID-19 PANDEMIC

**DOI:** 10.1093/geroni/igad104.1376

**Published:** 2023-12-21

**Authors:** Giorgio Di Gessa

**Affiliations:** University College London, London, England, United Kingdom

## Abstract

Volunteering, providing grandparental childcare, and provision of family care were quite common activities pre-pandemic among older people. However, during the pandemic, older people in particular were strongly advised to stay indoors, to work from home, and to avoid or at least limit interactions with people outside of their immediate household, including grandchildren and friends. As a result, evidence from the English Longitudinal Study of Ageing (ELSA) showed dramatic changes in the level of engagement in these socially productive activities. About 10% of grandparents stopped looking after grandchildren altogether during the first 9 months of the pandemic, with 22% reporting an overall decrease in the amount of grandchild care provided. Similarly, among those who were volunteering or caring for someone outside their household pre-COVID-19, almost two thirds decreased or stopped altogether engaging in these activities, with overall negative consequences for their mental health and wellbeing. To date, though, little is known on whether, in emerging from the current pandemic and as restrictions are lifted, social engagement among older people has resumed pre-COVID-19 pandemic levels and whether this is related to a rebound also in emotional wellbeing. Using data from ELSA collected in 2021/23 allows for an exploration of changes in grandparental childcare provision, caring, and volunteering since the COVID-19 pandemic, with a focus on levels of engagement among those older people who had stopped altogether these activities during the first months of the COVID-19 pandemic

